# Collagenase Clostridium Histolyticum Versus Percutaneous Needle Fasciotomy for Dupuytren’s Disease: A Systematic Review and Meta-Analysis

**DOI:** 10.3390/life15020259

**Published:** 2025-02-08

**Authors:** Ishith Seth, Vicki McClure, Bryan Lim, Roberto Cuomo, Richard J. Ross, Warren M. Rozen

**Affiliations:** 1 Faculty of Science, Medicine, and Health, Monash University, Melbourne, VIC 3004, Australia; 2 Department of Plastic and Reconstructive Surgery, Peninsula Health, Melbourne, VIC 3199, Australia; 3 Faculty of Science, Medicine, and Health, University of Siena, 53100 Siena, Italy

**Keywords:** Dupuytren’s contracture, percutaneous fasciotomy, collagenase, CCH, PNF

## Abstract

Minimally invasive treatments for Dupuytren’s disease (DD), such as percutaneous needle fasciotomy (PNF) and collagenase clostridium histolyticum (CCH), have become alternatives to open surgeries. This meta-analysis compared these treatments in terms of complications, patient satisfaction, clinical outcomes, and recurrence. Relevant studies up to June 2024 were identified through major databases, following PRISMA guidelines, and the study was registered on PROSPERO. Statistical analysis using Review Manager 5.4 found PNF had lower post-operative rates of oedema (RR = 0.15, 95% CI [0.09, 0.27], *p* < 0.00001), lymphadenopathy (RR = 0.09, 95% CI [0.02, 0.38], *p* = 0.0010), and pruritus (RR = 0.1, 95% CI [0.01, 0.73], *p* = 0.02) compared to CCH. However, there were no significant differences in skin tears, recurrence, reintervention, extension deficit, or residual flexion at metacarpal and proximal interphalangeal joints (*p* > 0.05). Patient-reported outcomes, including QuickDASH and URAM scores, also showed no significant differences. Eleven studies involving 1443 patients were analysed, and most were at a low-to-moderate risk of bias, as assessed using the Cochrane or Newcastle–Ottawa tools. While PNF showed fewer minor complications, overall clinical and patient-reported outcomes were comparable between the treatments. These findings highlight the need to tailor treatment choices to patient preferences and clinical context.

## 1. Introduction

Dupuytren’s disease (DD) is a fibroproliferative disease of the palmar fascia that results in a rigid subdermal cord and nodules, which eventually result in flexion contractures of the affected fingers [1] and finger pain, joint stiffness, and functional impairment of finger extension [1]. Open surgeries to excise fibrotic tissue were the mainstay for management. Still, minimally invasive methods have gained popularity over time, such as the use of collagenase clostridium histolyticum (CCH) and percutaneous needle fasciotomy (PNF), which is performed by 39% of members who are a part of the American Society for Surgery of the Hand [2].

In 1979, Lermusiaux and Debeyre first implemented a percutaneous needle as a substitute for surgical blades for lysing fibroproliferative bands [3]. This method, coined PNF, became popular due to its minimal invasiveness, low expense, favourable clinical outcomes, immediate results, and fast recovery. As such, it is one of the first recommended management options for elderly patients [2]. Despite this, its efficacy was found to be lower than that of open surgical intervention. Both methods are minimally invasive procedures that allow for regaining finger extension function. In addition, both methods have reported greater patient-reported outcome measures (PROMs) and fewer complication rates than open fasciotomies. Despite CCH and PNF having many advantages and favourable outcomes, the superiority of either treatment remains debatable.

With the cost of CCH treatment being substantially higher than PNF and normally requiring two courses of injection for efficacy, it is essential to distinguish the differences between PNF and CCH for clinicians and patients to make informed choices. Therefore, this systematic review and meta-analysis will summarise and analyse the clinical efficacy, durability, complication rates, and PROMs associated with PNF and CCH.

## 2. Methods

This meta-analysis was conducted according to the Cochrane Handbook of Systematic Reviews and the Preferred Reported Items for Systematic Review and Meta-Analysis (PRISMA). The protocol was registered in the Prospective Register of Systematic Reviews (PROSPERO).

### 2.1. Literature Search

Web of Science, Scopus, PubMed, Google Scholar, ClinicalTrials.gov, and Cochrane Library databases were searched for relevant articles up until June 2024. The search terms included “Dupuytrens” or “Dupuytren’s” or “Dupuytren contracture” or “Dupuytren disease” or “Dupuytren’s disease” and “collagenase” or “needle aponeurotomy” or “percutaneous needle fasciotomy” or “fasciectomy” or “Xiaflex” or “Fibromatosis contracture” or “CCH” or Xiapex” or “minimally invasive cord” or “manipulation extension procedure”, the terms were modified according to the recommendations of each database. No age, gender, and population filters were imposed. Duplicates were omitted via EndNote X9. A manual search of references for included studies and previous systematic reviews was also performed.

### 2.2. Eligibility Criteria

The inclusion criteria were as follows: (1) cohort, case–control studies, and clinical trial studies that compared CCH with PNF in treatment of DD; (2) reported outcomes included one or more of the following: complication, recurrence, and reintervention rate, residual flexion contracture, extension deficit, PROMs via Unité Rhumatologique des Affections de la Main (URAM) score and/or quick disabilities of the arm, shoulder and hand (QuickDASH) score for both CCH and PNF treatments; (3) articles with sufficient data to calculate odds ratio (OR) and 95% confidence interval (CI). Where possible, authors were contacted to request any missing information.

Exclusion criteria were as follows: study designs including reviews, case reports or series, letters to the editor, conference abstracts, animal studies, experimental or biomechanical studies, studies with absent full text or unextractable data, and studies not in the English language.

Two investigators (IS and BL) independently reviewed all studies and extracted relevant data. Studies marked for possible inclusion by either reviewer underwent dual, independent full-text review. Discrepancies were resolved by consensus and discussion with a third investigator (WMR).

### 2.3. Data Extraction

The subsequent data were extracted:

1. Demographics of patients and their outcomes, including age (years), gender (male and female), sample size, study design, joint number, finger number, follow-up period, type and number of complications, and clinical outcomes, including complication, recurrence, and reintervention rates, residual flexion contracture, extension deficit, URAM, and QuickDASH. The included studies defined recurrence as a loss of extension of the treated joint either by 20 or 30 degrees or more. The flexion and extension were reported as passive movements and the joint movements were measured and recorded.
2. The risk of bias in the included clinical trials was assessed using the Cochrane Collaboration risk of bias tool. Each domain was judged as ‘high risk of bias’, ‘low risk of bias’, or ‘unclear risk of bias’.
3. The quality of retrospective cohort studies was evaluated using the Newcastle–Ottawa Scale (NOS; www.ohri.ca/programs/clinical_epidemiology/oxford.asp, accessed on 12 August 2024). The studies were rated independently, and the mean of the resulting scores was recorded.

### 2.4. Data Synthesis and Analysis

For the analysis, Review Manager software (version 5.4.1) pooled estimate effects of the included study arms and a meta-analysis was performed. Dichotomous data were exhibited as risk ratio (RR) and 95% confidence interval (95% CI) using the Mantel–Haenszel statistical method, and continuous variables were exhibited as mean difference (MD) with 95% confidence interval (95% CI) using the inverse variance statistical method. Dichotomous outcomes were expressed as several outcomes, while continuous data were expressed as mean change (MD), either reported directly by the study or calculated as the difference between baseline and post-treatment values. Heterogeneity was assessed using Chi-squared (Q^2^) analysis and quantified by I-squared (I^2^) tests. I^2^ value over 50% and P of Q^2^ < 0.1 was considered statistically significant. Significant heterogeneity was addressed by a random-effects model and resolved by leave-out sensitivity analysis. Subgroup analysis on available follow-up duration was conducted whenever data were sufficient.

## 3. Results

### 3.1. Literature Search

**Figure 1:**
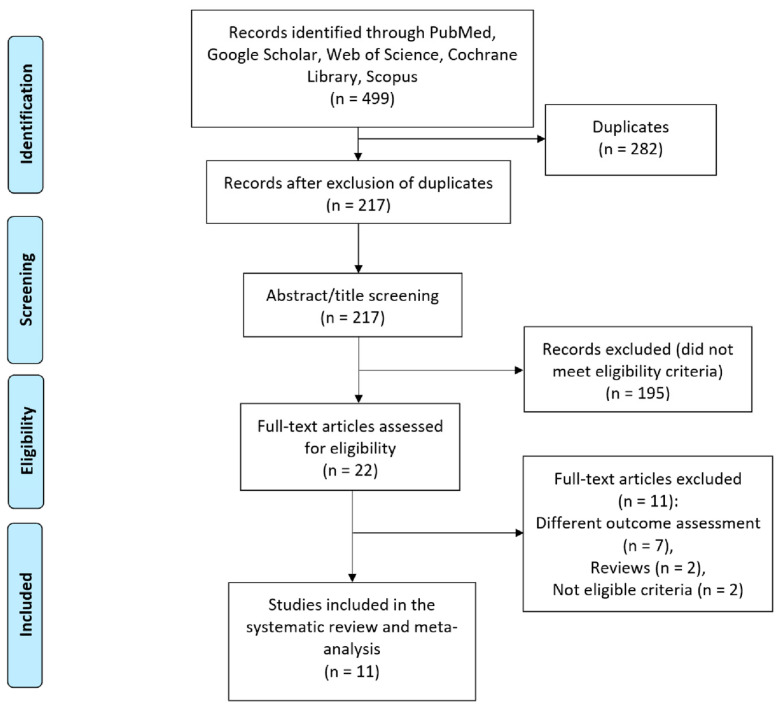
PRISMA flow diagram of selected studies.

The initial data retrieval process extracted 217 potentially eligible records. After screening for title and abstract according to inclusion and exclusion criteria, 22 studies were assessed for full-text screening. After further eligibility checking, 11 studies comparing CCH and PNF were included in this systematic review and meta-analysis. The study flow and selection process are summarised as a PRISMA flow diagram in Figure 1.

#### 3.1.1. Characteristics of Included Studies

A total of 11 studies comprising 1443 patients were included in the current systematic review and meta-analysis [4,5,6,7,8,9,10,11,12,13,14,15]. Eight studies were RCTs, two were retrospective cohorts, and only one was a prospective cohort design. From the pooled data, 1114 were males (77.2%), and the average age of the CCH cohort was 66.4 ± 8.1 years and 67 ± 7.9 years for PNF, as shown in Table 1. Types of complications and the post-operative clinical outcome of included MCP and PIP extension deficits, residual flexion, and recurrence/reoperation rates are reported in Table 2 and Table 3 and Supplementary Table S1. 

#### 3.1.2. Quality Assessment

**Figure 2:**
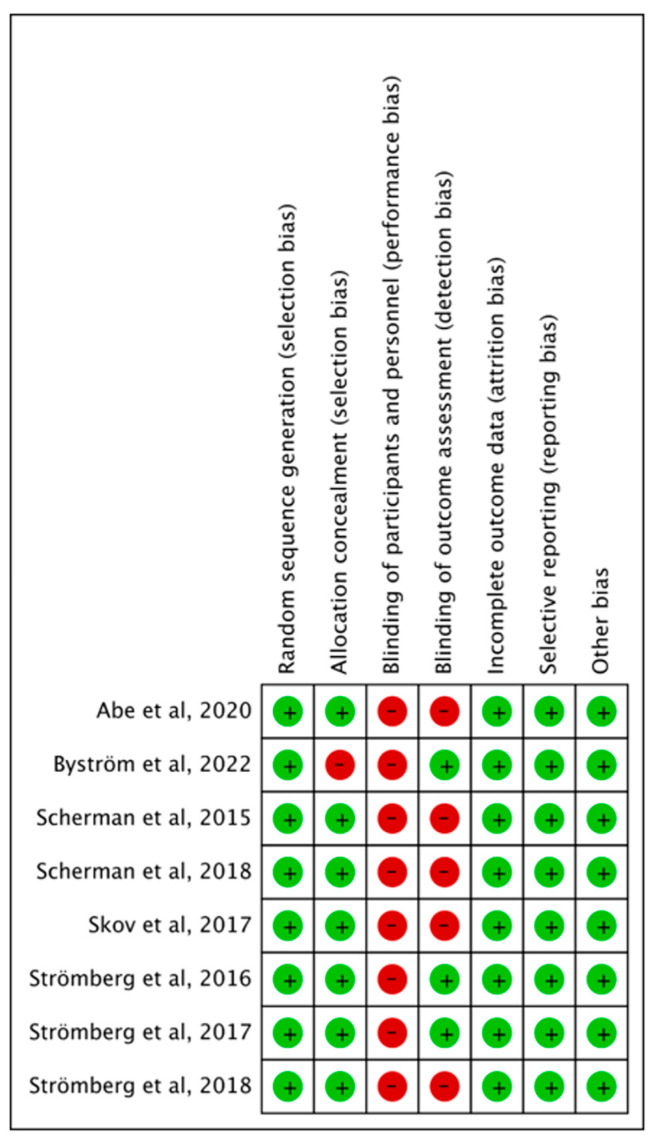
Summary of the quality assessment of the included randomised controlled trials. Green represents low risk of bias, yellow represents moderate risk of bias and red represents high risk of bias.

The quality of RCTs was evaluated by the Cochrane RoB-1 tool and illustrated in Figure 2. RCTs lacked double blinding in all studies but were otherwise considered high quality. Retrospective studies were evaluated using NOS (Supplementary Table S2); two [8,14] were considered high quality (Supplementary Table S3), while Nydick et al., 2013 [6] was considered moderate quality.

#### 3.1.3. Complications

**Figure 3:**
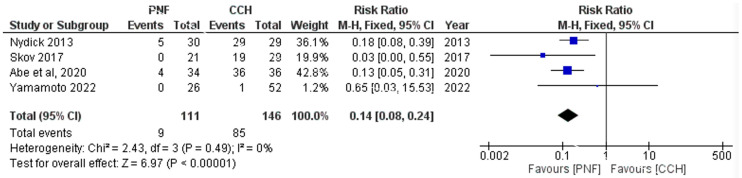
Forest plot comparing edema rate between collagenase clostridium histolyticum and percutaneous needle fasciotomy.

**Figure 4:**
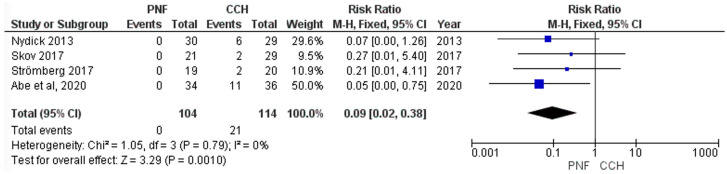
Forest plot comparing lymphadenopathy rate between collagenase clostridium histolyticum and percutaneous needle fasciotomy.

Edema was reported in four studies, lymphadenopathy in four studies and pruritus in two studies. Data for major complications were insufficient for meta-analysis. Overall, the post-operative complications were significantly lower in PNF compared with CCH, with CCH having higher post-operative edema (RR = 0.15 [0.09, 0.27], *p* < 0.00001), lymphadenopathy (RR = 0.09 [0.02, 0.38], *p* = 0.001), and pruritus (RR = 0.1 [0.01, 0.73], *p* = 0.02). No significant heterogeneity was detected between the included studies reporting and edema (*p* = 0.49, I^2^ = 0%), lymphadenopathy (*p* = 0.79, I^2^ = 0%), and pruritus (*p* = 0.59, I^2^ = 0%), (Figure 3 and Figure 4 and Supplementary Figure S1, respectively).

No significant differences were detected between CCH and PNF for skin tears (RR = 0.86 [0.54, 1.39], *p* = 0.55) and the results were homogenous (I^2^ = 37%, *p*= 0.19, Supplementary Figure S2). No major complications (tendon rupture, nerve, or vessel injury) were reported in either treatment group.

#### 3.1.4. Recurrence Rate and Reintervention Rate

The reintervention rate was reported by seven studies. No statistically significant differences were found for reintervention rates between groups for one to two years (RR = 1.03 [0.25, 4.22], *p* = 0.96) and five years (RR = 1.06 [0.89, 1.26], *p* = 0.51). Recurrence rates were also insignificant between one and two years (RR = 0.87 [0.38, 2.01], *p* = 0.75) and five years (RR = 1.11 [0.68, 1.80], *p* = 0.68). No statistically significant heterogeneity was detected between included studies for recurrence rate (I^2^ = 0%, *p* = 0.50 *p* = 0.73 for 1–2 and 5 years, respectively), or reintervention rate (*p* = 0.28; I^2^ = 13%), while heterogeneity was evident at 2 to 3 years (*p* = 0.009; I^2^ = 85%, Supplementary Figures S3 and S4).

#### 3.1.5. Extension Deficit

Extension at the MCPJ pre- and post-operation was reported by nine studies. Extension deficits at the MCPJ were not statistically different between groups within the first 3 months (MD = 0.08 [−6.73, 6.88], *p* = 0.98), 3–6 months (MD = −0.12 [−3.35, 3.10], *p* = 0.94) and were homogenous (I^2^ = 0%, *p* = 0.98 and 0.50, respectively). For follow-up between 18 and 36 months, differences were not statistically different (MD = 3.19 [−8.46, 14.84] *p* = 0.59); I^2^ = 0%) and heterogeneity was present (*p* = 0.05; I^2^ = 75%). Extension at the PIP joint was examined pre- and post-operatively by 5 studies in total and no statistically significant differences were detected between CHH and PNF in all subgroups (1–3 months; MD = 3.44 [−5.21, 12.09], *p* = 0.44; 3–6 months MD = 2.23 [−4.54, 9.00], *p* = 0.52; and 18 to 36 months MD = 6.72 [−6.29, 19.72], *p* = 0.31). Studies were homogenous for the 1-to-3- and 3-to-6-month subgroups (*p* = 0.20, I^2^ = 37%; *p* = 0.20, I^2^ = 33%, respectively), while this was heterogenous for the 18 to 36 months group (*p* = 0.009, I^2^ = 79%), as shown in Supplementary Figures S5 and S6.

#### 3.1.6. Residual Flexion Contracture

Residual flexion contractures at MCP and PIP were reported by two studies. At the MCP, MD was not significant between CCH and PNF (MD = 0.79 [−2.98, 4.55] *p* = 0.68, Supplementary Figure S5) and studies were homogenous (*p* = 0.31, I^2^ = 3%). At the PIP, MD was not significantly different (MD = −0.28 [−4.21, 3.64], *p* = 0.89, Supplementary Figure S5) and studies were homogenous (*p* = 0.76, I^2^ = 0%), as shown in Supplementary Figures S7 and S8.

#### 3.1.7. Patient-Reported Outcome Measures (URAM and QuickDASH Scores)

In total, five studies reported QuickDASH scores. No statistically significant differences were detected between the CCH and PNF groups in both subgroups (MD = 2.73 [−6.41, 11.87], *p* = 0.56), (MD = 0.25 [−1.92, 2.43], *p* = 0.82) after one year (*p* = 0.05, I^2^ = 74%) and two years (*p* = 0.14, I^2^ = 50%). The URAM score was reported by five studies in total and no significant difference between CCH and PNF was found (MD = 0.18 [−1.61, 1.97], *p* = 0.84) after one year and two years (MD = 0.41 [−0.84, 1.65], *p* = 0.52). The pooled studies were homogenous (*p* = 0.61; I^2^ = 0%) and (*p* = 0.14, I^2^ = 50%), following one and two years, respectively (Supplementary Figures S9 and S10).

## 4. Discussion

This systematic review and meta-analysis provide a comprehensive comparison of Collagenase Clostridium Histolyticum and Percutaneous Needle Fasciotomy in the management of Dupuytren’s disease. While both treatments demonstrated comparable efficacy in improving extension deficits, reducing residual flexion contractures, and maintaining low recurrence and reintervention rates, CCH was associated with a higher incidence of minor complications, including edema, lymphadenopathy, and pruritus. Despite these differences, no major complications were reported for either modality, underscoring the safety and effectiveness of both minimally invasive techniques. These findings highlight the critical role of patient-specific considerations, such as cost, complication tolerance, and treatment goals, in guiding clinical decision-making for DD management.

This study concluded a higher overall rate of complications with CCH although the profiles of these were minor. Skov et al. reported a significantly higher rate of complications with CCH; however, complications consisted of digital neuropraxia and edema which do not usually disturb function post-treatment or normally cause permanent morbidity. Nydick et al. also reported a significantly higher average complication rate following CCH (55%) compared with PNF (15%) but none were major and were all resolved without intervention within 8 to 10 days [15,17]. CCH works by cleaving collagen strands through enzymatic degradation and cord rupture, subsequently improving digital contracture. CCH’s higher complication rate profile may be explained by inflammatory events following the injection of collagenase where the production of pro-inflammatory mediators may result in pain, edema, ecchymosis, pruritis, and lymphadenopathy. Because of this, there are distinct differences between the timelines for both treatments, with CCH allowing for the treatment of two joints or an additional ray at one setting which may be administered up to three times per cord every 4 weeks [18] with no limit on the contractures performed at one time in a single procedure for PNF, allowing for quicker outcomes in PNF. In addition, CCH is around USD 5000 versus USD 500 for PNF^8^. Therefore, while the findings of our study suggest CCH is inferior because of its complication rate, in the context of complication types, none are acquired long-term or cause significant morbidity.

Given the absence of major complications for CCH and PNF and a minor complication profile that is likely to improve without intervention in both groups, these results endorse both treatment modalities for DD [4,14,19]. Alternative surgical treatments with similar efficacy confer higher surgical and anesthetic complication profiles, with invasive open DD repairs having major complication rates ranging between 1 and 23% (tendon rupture, arterial injury, and nerve injury) and also carrying an unplanned amputation rate of 8% when digital arteries are accidentally dissected. Whilst suboptimal, these complications risk come with the likelihood of better improvements in extension function. For example, RCTs comparing LF with PNF demonstrated PNF had greater residual extension deficits (79% versus 63%), and higher rates of recurrence (85% versus 21%), but with lower rates of complications (5% versus 0%) [20,21]. Therefore, the benefits of invasive and minimally invasive procedures need to be weighed alongside their long-term outcomes and complications. Based on Denkler et al. 2022 and the current results, a treatment algorithm ladder could be considered in future studies for the treatment pathway of DD [19]. Herein, PNF or CCH could be considered an initial treatment, and if recurrence occurs more than two years after the initial treatment, it could be repeated. A more invasive treatment may be considered if the recurrence is less than 2 years [19].

The popularity of minimally invasive treatments of DD has increased because of their efficacy, availability, and feasibility outside the hospital setting. This meta-analysis reports that CCH and PNF groups did not significantly differ in recurrence or reintervention rates, although results were heterogeneous. For example, Scherman et al. compared PNF with CCH in 50 patients with metacarpal phalangeal joint (MCPJ) DD over a 3-year follow-up and found the success rate was 100% and 89%, respectively, the recurrence rate was 68% and 83%, respectively, and no difference in retreatment rate was observed [13]. In contrast, a study by Van Rijssen and Werker with a similar study design to Scherman et al. but with a larger PIPJ cohort found recurrence rates of DD for PNF after 33 months were 65%, which is much higher than the current study’s (14.6%) [13], although reintervention rates for PNF (42%) were comparable to the current study (43.6%). The network meta-analysis by Nann et al. further supported these findings, showing that at 2–5 years post-treatment, patients with PIPJ contractures had significantly worse outcomes than those with MCPJ involvement, regardless of treatment modality [22]. The recurrence rate for PIPJ after PNF was found to be 9.10 times higher than after fasciectomy (*p* < 0.05), while MCPJ recurrence remained lower across all interventions. Additionally, total passive extension deficit (TPED) was significantly greater in PIPJ compared with MCPJ following CCH and PNF, reinforcing the need for surgical intervention in severe PIPJ cases. These results are aligned with the literature that suggests CCH and PNF may have less long-term efficacy for PIP than MCP contractures [23,24,25] with the CORDLESS study finding the recurrence rate was double in PIPJ compared with MCPJ, notably for severe PIPJ. PIPJ is known to be recalcitrant to DD therapies [13,23], although the literature is not conclusive, Van Rijssen’s and Werker et al.’s higher recurrence in PIPJs adds to this hypothesis. Future studies should stratify recurrence according to the type of joints affected to better understand this association.

The comparative efficacy of CCH and PNF was not significantly different at MCPJs or PIPJs across the follow-up subgroup analyses. While no temporal associations were established, the CORD trial reported a 79% and 71% improvement after 30 days in MCPJs and PIPJs using CCH, respectively. PNF had similar results according to Rahr et al., who found early improvements of 71%, 75%, 75%, and 65% for Tubiana DD classifications I-IV, respectively, after only six weeks of treatment. Skov et al. observed that CCH had worse clinical improvement than PNF (8% versus 3%) and complications (93% versus 24%) after two years in PIP joints where clinical improvement was considered a decrease of 50% or more from baseline contracture. At three years, 49% of PNF and 47% of CCH had maintained their post-operative results and were considered clinically improved.

In our study, the URAM scale and QuickDASH scores showed improvements following both treatments, but no statistically significant differences between CCH and PNF groups were observed. PROMs, such as the URAM scale and QuickDASH questionnaire, are less commonly used for DD evaluation than biometric measurements, such as contracture measurements or extension deficits, and this is likely because there is currently no consensus on which PROM is best for DD [11,13,16,26]. Rodrigues et al. noted that URAM did not accurately capture patient problems in 55% of patients; however, Bernabé et al. found it correlated well with VAS scores and other disability measures more than QuickDASH [27,28]. Considering PROMs are extremely important for assessing quality of life, future studies should focus on bridging consensus on other measures, such as the Michigan Hand Outcomes Questionnaire, for correlation with post-operative improvements [12,29].

This study analysed 1443 patients across 11 studies, incorporating subgroup analyses and predominantly homogeneous outcomes, with most of the included studies being randomised controlled trials. These methodological strengths enhance the reliability of the findings and build upon the insights provided by previous systematic reviews [30]. However, several limitations should be considered. Heterogeneity in certain outcomes could not be fully resolved through subgroup or sensitivity analyses. Additionally, the lack of consensus on the most effective patient-reported outcome measures for Dupuytren’s disease, such as URAM and QuickDASH, may limit the relevance of these tools in future evaluations. Limited data further constrained the analysis, preventing stratification based on the specific fingers affected. Other important confounders, including study design, sex, and treatment type, whether primary or for recurrence, were not accounted for in the stratification. Furthermore, the exclusion of invasive surgical techniques from this analysis limits the ability to directly compare recurrence rates and contracture correction between minimally invasive and surgical treatments. These outcomes are highly relevant to patients and play a critical role in treatment decision-making. Future research should aim to directly compare surgical and minimally invasive interventions to provide a more comprehensive understanding of their respective advantages and limitations.

Future studies should focus on developing and implementing universally standardised patient-reported outcome measures to improve the consistency and comparability of functional outcomes in Dupuytren’s disease treatment. Currently, there is no consensus on the optimal PROM for evaluating treatment efficacy, with scales such as URAM and QuickDASH frequently used but not uniformly validated for this condition. Incorporating more comprehensive tools, such as the Michigan Hand Outcomes Questionnaire, which captures broader functional and quality-of-life metrics, could enhance the assessment of patient-centered outcomes. Standardised methodologies should also include uniform definitions of recurrence, reintervention, treatment success, and long-term follow-up protocols to ensure consistent reporting. Future randomised controlled trials comparing CCH, PNF, and surgical interventions should aim to stratify recurrence rates based on joint involvement (MCPJ vs. PIPJ), severity, and prior treatments to refine treatment algorithms. By addressing these methodological gaps, future research can provide more clinically relevant insights, optimising patient care and guiding evidence-based decision-making in Dupuytren’s disease management.

## 5. Conclusions

This meta-analysis found no significant differences between PNF and CCH for improving extension deficits, residual flexion, recurrence or reintervention rates, and PROMs. A higher minor complication rate in CCH for edema, lymphadenopathy, and pruritus was noted, albeit these are minor and short-term. Further studies should compare PNF and CCH with invasive surgical management to clarify the value of going under the knife for DD management.

## Figures and Tables

**Table 1:** Characteristics of the included studies.

Study	Study Design	Intervention Groups	Total Patients	Male Number (% of Total)	Average Age (years)	Fingers Affected ^a^	Joints Affected ^b^	Follow-Up Period (years)
[12]	RCT	CCH	36	36 (100)	69.6	NR	46	3
		PNF	34	31 (86)	67.2	NR	48	
[4]	RCT	CCH	78	65 (83)	65 (±8.1)	78	NR	5
		PNF	78	68 (87)	65 (±9.1)	78	NR	
[8]	Retrospective cohort	CCH	153	115 (75)	65.8	NR	NR	5
		PNF	367	314 (86)	63.8	NR	NR	
[16]	Retrospective cohort	CCH	29	24 (85)	67 (±10)	NR	34	0.5
		PNF	30	22 (75)	66 (±10)	NR	50	
[5]	RCT	CCH	38	36 (95)	67	NR	40	1
		PNF	45	36 (80)	67	NR	46	
[13]	RCT	CCH	38	36 (95)	67	NR	40	3
		PNF	45	36 (80)	67	NR	46	
[7]	RCT	CCH	29	20 (69)	62 (58–66) ^c^	NR	NR	2
		PNF	21	17 (81)	67 (64–70) ^c^	NR	NR	
[10]	RCT	CCH	69	56 (81)	66	70	NR	1
		PNF	71	63 (89)	69	71	NR	
[9]	RCT	CCH	20	17 (85)	65	20	NR	1
		PNF	19	17 (89)	67	19	NR	
[11]	RCT	CCH	78	65 (83)	65	78	NR	2
		PNF	78	68 (87)	68	78	NR	
[14]	Prospective observation study	CCH	52	50 (96)	71 ± 8.9	3.8 ± 1 ^1^	2.2 ± 0.5 ^2^	0.5
		PNF	26	25 (96)	70 ± 7.6	4.2 ± 0.9 ^1^	2.2 ± 0.4 ^2^	

**Abbreviations:** CCH; collagenase clostridium histolyticum, NR; not reported, PNF; percutaneous needle fasciotomy, RCT; randomised controlled trial, STD; standard deviation. ^a^ Total joint involved in the treatments. ^b^ Total finger involved in the study treatments. ^c^ 95% confidence interval. ^1^ Total affected finger per patient (mean ± STD). ^2^ Total affected joints per patient (mean ± STD).

**Table 2:** Minor complications of the included studies.

Study	Groups	Minor Complications			
		Edema, n (%)	Pruritus, n (%)	Skin Tear, n (%)	Lymphadenopathy, n (%)
[12]	CCH (n = 36)	36 (100)	NA	9 (25)	11 (30.6)
	PNF (n = 34)	4 (11.8)	NA	6 (17.6)	0 (0)
[16]	CCH (n = 29)	29 (100)	7 (24.1)	10 (34.5)	6 (20.7)
	PNF (n = 30)	5 (16.7)	0 (0)	15 (50)	0 (0)
[7]	CCH (n = 29)	19 (66)	3 (10.3)	6 (20.7)	2 (6.8)
	PNF (n = 21)	0 (0)	0 (0)	2 (9.5)	0 (0)
[14]	CCH (n = 52)	1 (2%)	NA	5 (10%)	NA
	PNF (n = 26)	0 (0%)	NA	0 (0%)	NA

**Abbreviations:** CCH; collagenase clostridium histolyticum, NA; not available, PNF; percutaneous needle fasciotomy.

**Table 3:** Reintervention and recurrence rates of the included studies.

Study	Groups	Recurrence, n (%)	Reintervention, n (%)
[4]	CCH (n = 71)	NA	36 (51)
	PNF (n = 72)	NA	33 (46)
[8]	CCH (n = 153)	NA	74 (48)
	PNF (n = 367)	NA	224 (61)
[13]	CCH (n = 35)	NA	4 (11)
	PNF (n = 45)	NA	11 (24)
[10]	CCH (n = 67)	1 (2)	NA
	PNF (n = 71)	1 (2)	NA
[11]	CCH (n = 76)	10 (13)	NA
	PNF (n = 76)	9 (12)	NA
[13]	CCH (n = 36)	12 (33)	NA
	PNF (n = 40)	17 (35)	NA
[14]	CCH (n = 52)	8 (18%)	NA
	PNF (n = 26)	2 (9.5%)	NA

**Abbreviations:** CCH; collagenase clostridium histolyticum, NA; not available, PNF; percutaneous needle fasciotomy.

## Data Availability

Data for this study is available upon reasonable request from the corresponding author.

## Supplementary Materials

The following supporting information can be downloaded at: www.mdpi.com/article/10.3390/life15020259/s1, Table S1. Quality assessment of retrospective cohort studies; Table S2. Quality assessment of prospective observational study; Figure S1. Forest plot comparing pruritus rate between collagenase clostridium histolyticum and percutaneous needle fasciotomy; Figure S2. Forest plot comparing skin tear rate between collagenase clostridium histolyticum and percutaneous needle fasciotomy; Figure S3. Forest plot comparing reintervention rate between collagenase clostridium histolyticum and percutaneous needle fasciotomy; Figure S4. Forest plot comparing recurrence rate between collagenase clostridium histolyticum and percutaneous needle fasciotomy; Figure S5. Forest plot comparing collagenase clostridium histolyticum and percutaneous needle fasciotomy in terms of Extension at MCP; Figure S6. Forest plot comparing collagenase clostridium histolyticum and percutaneous needle fasciotomy in terms of Extension at PIP; Figure S7. Forest plot comparing collagenase clostridium histolyticum and percutaneous needle fasciotomy in terms of Flexion at MCP; Figure S8. Forest plot comparing collagenase clostridium histolyticum and percutaneous needle fasciotomy in terms of Flexion at PIP; Figure S9. Forest plot comparing collagenase clostridium histolyticum and percutaneous needle fasciotomy in terms of URAM score; Figure S10. Forest plot comparing collagenase clostridium histolyticum and percutaneous needle fasciotomy in terms of QuickDASH score.

## Abbreviations

The following abbreviations are used in this manuscript:

DD	Dupuytren’s Disease
PNF	Percutaneous needle fasciotomy
CCH	Collagenase clostridium histolyticum
OR	Odds Ratio
CI	Confidence Interval
IS	Ishith Seth (Author)
BL	Bryan Lim (Author)
WMR	Warren Rozen (Author)
RR	Risk Ratio
MD	Mean Difference
MCP	Metacarpophalangeal
PIP	Proximal interphalangeal
PROM	Patient reported outcome measure
MCPJ	Metacarpal phalangeal joint
NR	Not reported
RCT	Randomised Control Trial
STD	Standard Deviation
