# Supplementary material for: Collagenase Clostridium Histolyticum Versus Percutaneous Needle Fasciotomy for Dupuytren’s Disease: A Systematic Review and Meta-Analysis
Source: Life (Basel). 2025 Feb 8;15(2):259. doi: 10.3390/life15020259 (PMC11856867; doi:10.3390/life15020259)
Supplement: Supplementary file 1 [file life-15-00259-s001.zip › life-3439112-supplementary.pdf]

Supplementary Table S1: Quality assessment of retrospective cohort studies.

Study	Adequate definition of the cases	Representation of the cases	Selection of controls	Definition of controls	Comparability of cases and controls based on the design or analysis	Ascertainment of exposure	Same method of ascertainment of exposure for cases and controls	Non-response rate	Total
Leafblad et al., 2019	A (+1*)	B (0)	B (0)	A (+1*)	A (+1*)	A (+1*)	A (+1*)	A (+1*)	6
Nydick et al., 2013	A (+1*)	B (0)	B (0)	A (+1*)	A (+1*)	A (+1*)	A (+1*)	A (+1*)	6

*Score star

Supplementary Table S2: Quality assessment of prospective observational study.

Study/Item	Representativeness of the exposed cohort	Selection of the non-exposed cohort	Ascertainment of exposure	Demonstration of the outcome of interest at start of study	Comparability of cohorts based on the design or analysis	Assessment of outcome	Was follow-up long enough for outcomes to occur	Adequacy of follow up of cohorts	Total[a]
Yamamoto et al., 2022.	A (+1*)	A (+1*)	A (+1*)	A (+1*)	A (+1*)	D (0)	A (+1*)	B (+1*)	7

* Score star

[a] Total score (Stars)

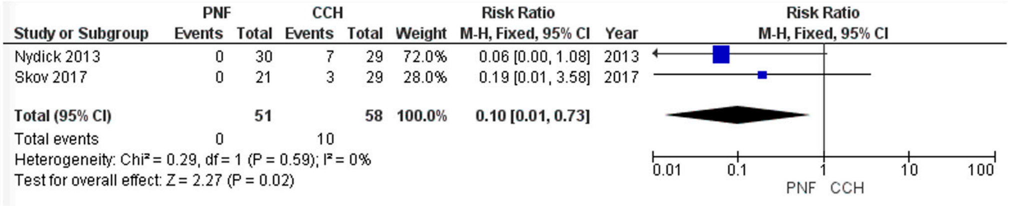

Supplementary Figure S1. Forest plot comparing pruritus rate between collagenase clostridium histolyticum and percutaneous needle fasciotomy.

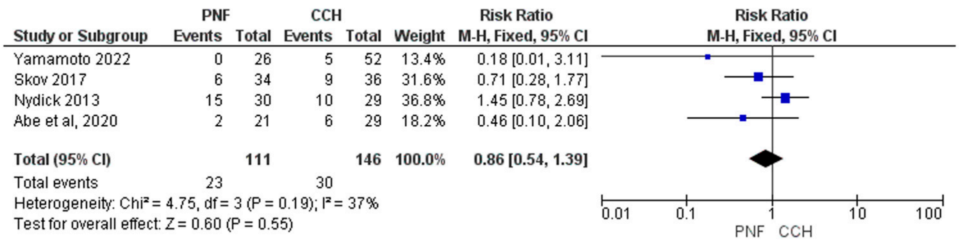

Supplementary Figure S2. Forest plot comparing skin tear rate between collagenase clostridium histolyticum and percutaneous needle fasciotomy.

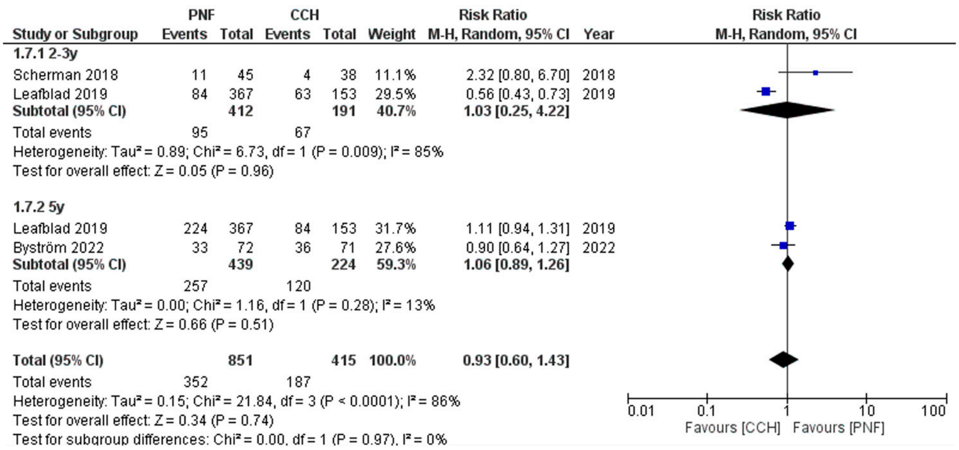

Supplementary Figure S3. Forest plot comparing reintervention rate between collagenase clostridium histolyticum and percutaneous needle fasciotomy.

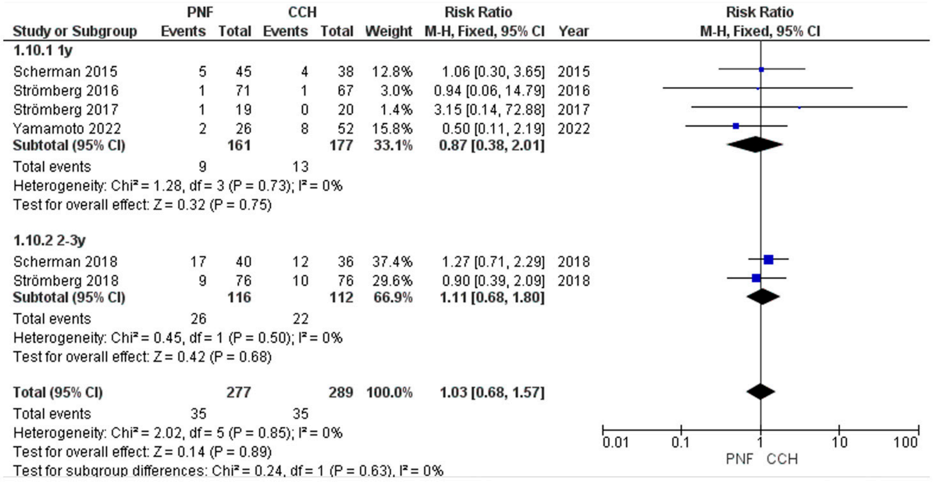

Supplementary Figure S4. Forest plot comparing recurrence rate between collagenase clostridium histolyticum and percutaneous needle fasciotomy.

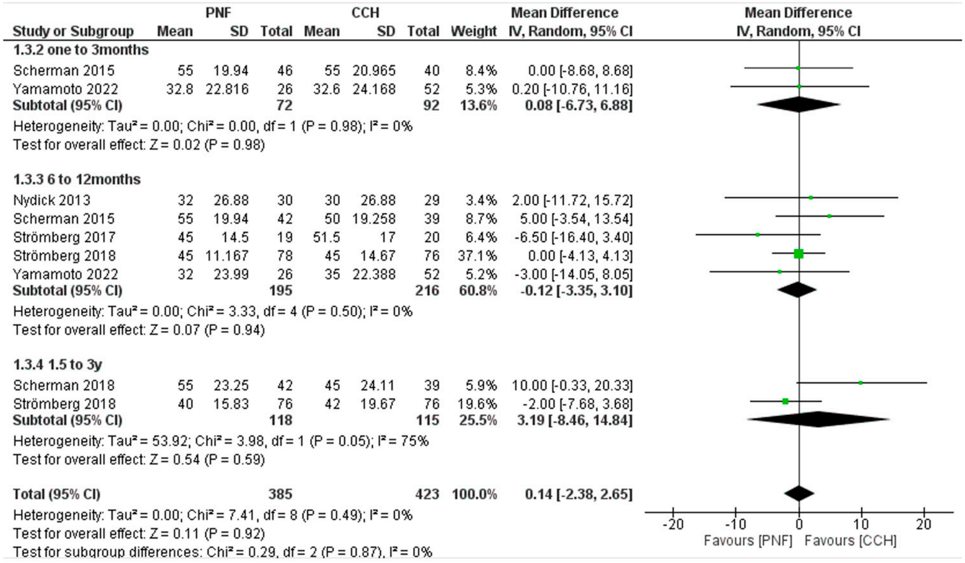

Supplementary Figure S5. Forest plot comparing collagenase clostridium histolyticum and percutaneous needle fasciotomy in terms of extension at MCP.

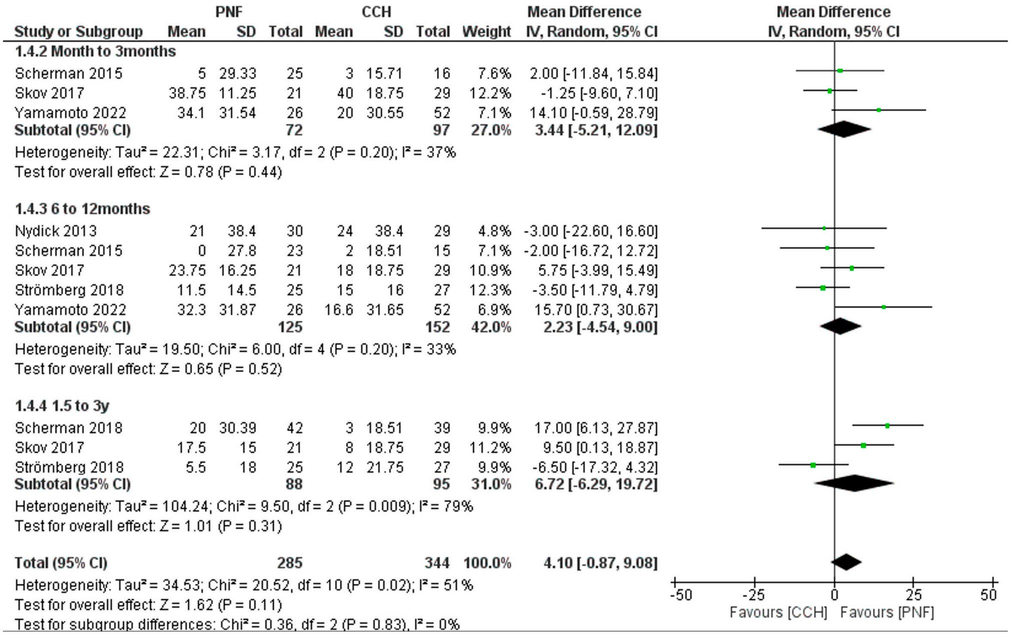

Supplementary Figure S6. Forest plot comparing collagenase clostridium histolyticum and percutaneous needle fasciotomy in terms of extension at PIP.

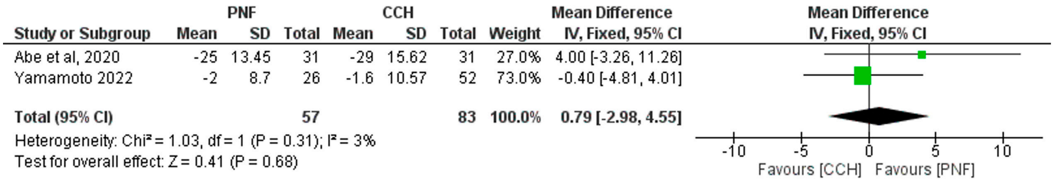

Supplementary Figure S7. Forest plot comparing collagenase clostridium histolyticum and percutaneous needle fasciotomy in terms of flexion at MCP.

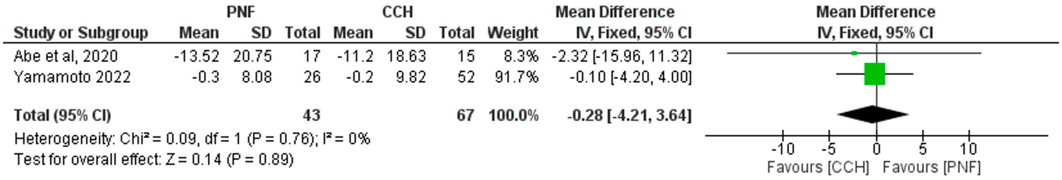

Supplementary Figure S8. Forest plot comparing collagenase clostridium histolyticum and percutaneous needle fasciotomy in terms of flexion at PIP.

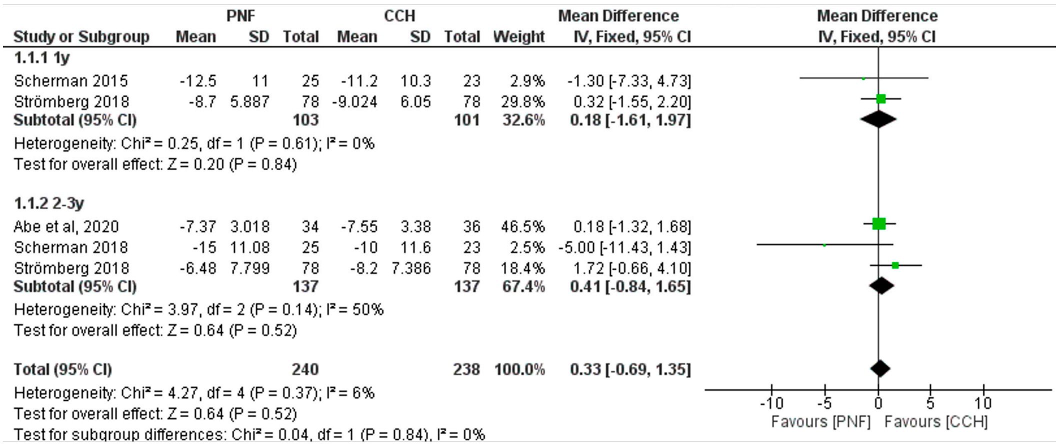

Supplementary Figure S9. Forest plot comparing collagenase clostridium histolyticum and percutaneous needle fasciotomy in terms of URAM score.

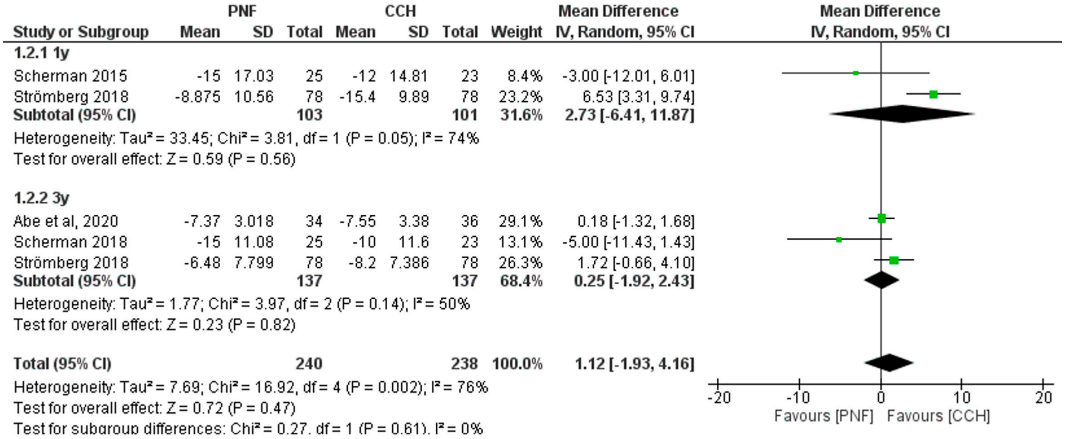

Supplementary Figure S10. Forest plot comparing collagenase clostridium histolyticum and percutaneous needle fasciotomy in terms of QuickDASH score.
